# The combined vaccination protocol of DNA/MVA expressing Zika virus structural proteins as efficient inducer of T and B cell immune responses

**DOI:** 10.1080/22221751.2021.1951624

**Published:** 2021-07-15

**Authors:** Patricia Pérez, Miguel A. Martín-Acebes, Teresa Poderoso, Adrián Lázaro-Frías, Juan-Carlos Saiz, Carlos Óscar S. Sorzano, Mariano Esteban, Juan García-Arriaza

**Affiliations:** a Department of Molecular and Cellular Biology, Centro Nacional de Biotecnología (CNB), Consejo Superior de Investigaciones Científicas (CSIC), Madrid, Spain; b Department of Biotechnology, Instituto Nacional de Investigación y Tecnología Agraria y Alimentaria (INIA), Consejo Superior de Investigaciones Científicas (CSIC), Madrid, Spain; c Molecular Virology Group, Department of Experimental and Health Sciences, Universitat Pompeu Fabra, Barcelona, Spain; d Biocomputing Unit, Centro Nacional de Biotecnología (CNB), Consejo Superior de Investigaciones Científicas (CSIC), Madrid, Spain

**Keywords:** ZIKV, DNA, MVA, vaccines, prime/boost, T-cell immune response, neutralizing antibodies, mice

## Abstract

Zika virus (ZIKV) is a mosquito-borne pathogen with public health importance due to the high risk of its mosquito vector dissemination and the severe neurological and teratogenic sequelae associated with infection. Vaccines with broad immune specificity and control against this re-emerging virus are needed. Here, we described that mice immunized with a priming dose of a DNA plasmid mammalian expression vector encoding ZIKV prM-E antigens (DNA-ZIKV) followed by a booster dose of a modified vaccinia virus Ankara (MVA) vector expressing the same prM-E ZIKV antigens (MVA-ZIKV) induced broad, polyfunctional and long-lasting ZIKV-specific CD4^+^ and CD8^+^ T-cell immune responses, with high levels of CD4^+^ T follicular helper cells, together with the induction of neutralizing antibodies. All those immune parameters were significantly stronger in the heterologous DNA-ZIKV/MVA-ZIKV immunization group compared to the homologous prime/boost immunizations regimens. Collectively, these results provided an optimized immunization protocol able to induce high levels of ZIKV-specific T-cell responses, as well as neutralizing antibodies and reinforce the combined use of DNA-based vectors and MVA-ZIKV as promising prophylactic vaccination schedule against ZIKV.

## Introduction

Zika virus (ZIKV) is a mosquito-borne pathogen from the family *Flaviviridae* and the genus *Flavivirus*. ZIKV was discovered in Uganda in 1947, but was confined for the first 60 years to an equatorial zone across Africa and Asia, where only sporadic outbreaks of infections were reported over the next few decades. The virus emerged in 2007 causing an outbreak in the Yap Island of Federated States of Micronesia, with the majority of symptomatic patients exhibiting fever, rash, and arthritis/arthralgia. A larger outbreak of the virus followed in French Polynesia and other Pacific Islands in late 2013, where, in addition to the above-described symptoms, conjunctivitis was also noted. The virus reached Latin America in 2015, and disseminated further to North America in 2016, with 500,000–1,500,000 suspected cases of ZIKV infection reported in the Americas, and more than 4300 cases of microcephaly [1]. As a consequence, the WHO declared the Public Health Emergency of International Concern from 1 February to 18 November 2016. The number of incidences in the Americas and the world has waned significantly after 2017 [2]. However, the virus still is circulating and its behaviour could be unpredictable due to its potential for re-emergence [3].

ZIKV is transmitted to humans primarily through the bite of infected mosquitoes from genus *Aedes*, mainly by *A. albopictus* and *A. aegypti*, both widely distributed throughout the tropical and subtropical regions of the world, with the habitat of *A. albopictus* extending further into cool temperate regions [1]. Based on model predictions, in the worst-case scenario, over 1.3 billion new people could face suitable transmission temperatures for ZIKV by 2050 [4]. Furthermore, ZIKV can also be transmitted from mother to child during pregnancy or spread through sexual contact, breastfeeding, or blood transfusion [1,2]. The multiple modes of ZIKV transmission make it difficult to develop control strategies against the pathogen.

In most cases, ZIKV infection causes no symptoms or only a mild self-limiting illness, but recent epidemiological studies derived from outbreaks in 2007 and 2015–2016 linked ZIKV infection to a rising number of concerning severe neurological diseases, including Guillain-Barré syndrome and severe congenital conditions such as brain calcifications, arthrogryposis, ophthalmologic alterations, spinal deformities, and microcephaly in neonates [1,2].

ZIKV contains a linear positive sense, single-stranded RNA genome of approximately 11 kb in length, which encodes a single open reading frame that is translated to produce a large polyprotein of 3423 amino acids that is cleaved by viral and cellular proteases into 10 individual proteins: three structural proteins located at the N-terminal region that form the infectious virion [the capsid (C) protein, the viral membrane (M) protein [a cleavage product of the prM protein] and the envelope (E) protein], and seven non-structural proteins, located at the C-terminal region, which are involved in viral replication (NS1, NS2A, NS2B, NS3, NS4A, NS4B, and NS5) [1]. Serologic and genome analyses suggest the existence of only one single serotype with three distinct genetic lineages: East African (that includes the first isolate from Uganda, MR766), West African, and Asian-American (including all contemporary strains from Asia, Oceania, and the Americas) [5].

The development of a safe and efficacious vaccine against ZIKV is critical given the high risk of *A. albopictus* dissemination and the severe neurological and teratogenic sequelae associated with ZIKV infection. Although, in recent years several vaccine candidates against ZIKV have been developed, and some of them entered in phase I or II clinical trials, there are no approved vaccines yet to prevent ZIKV infection [6]. One of the most promising vaccine vectors is the poxvirus modified vaccinia virus Ankara (MVA) that has been widely used as a recombinant vaccine vector against several infectious diseases and cancer [7–9]. and have numerous characteristics that make MVA an excellent vaccine candidate: (a) the packing flexibility of the genome, which allows the insertion of up to 25 kbp of foreign DNA without loss of infectivity, (b) the lack of persistence or genomic integration in the host due to their cytoplasmic replication, (c) the ability to induce both antibody and cytotoxic T cell immune responses against the heterologous antigens with long-lasting immunity after a single inoculation, (d) the stability of freeze-dried vaccine, (e) its ease of manufacture and administration and (f) the low prevalence of anti-vector immunity in the global population due to the interruption of smallpox vaccination after the WHO declared its eradication in 1980 [8,9]. The efficacy of recombinant MVA vectors in developing antigen-specific immune responses is due to the expression of gene products within cells that are efficiently presented by both MHC class I and class II molecules, leading to the activation of CD4^+^ and CD8^+^ T cells and the induction of potent antibody responses, which promotes robust anti-viral responses that make MVA to act as an adjuvant itself [10,11].

We have previously described the generation of an MVA-based ZIKV vaccine candidate expressing prM-E ZIKV proteins, termed MVA-ZIKV [12]. This candidate enabled the production of virus-like particles (VLPs) and induced ZIKV-specific CD8^+^ T cells and neutralizing antibodies in immunized mice, controlling ZIKV replication in a challenged mouse model [12]. Although MVA-ZIKV was highly immunogenic and protective in susceptible mice models at short timepoints, novel vaccine candidates and/or immunization approaches that could improve the magnitude, and durability of the ZIKV-specific immune responses are desirable. One of the most commonplace methods of immunization used to improve the immune responses generated by MVA-based vaccines is to follow a multiple dose prime/boost strategy, because priming with a different vector impairs the generation of anti-MVA responses able to abrogate boosted immune responses to the antigen encoded in the recombinant MVA vector [13]. The first description of the advantage of combined vectors to enhance specific immune responses and efficacy against a pathogen was work published with influenza and poxvirus vectors expressing a malaria CS antigen, showing that the heterologous combination was superior to homologous vectors, as well as it pointed out the relevance for immunogenicity and protection of the order of vector delivery [14]. The use of heterologous prime/boost protocols that combine an MVA vector with other vaccine agent such as DNA, can improve the antigen-specific immune responses in different animal models [7,15,16]. Furthermore, the use of heterologous prime/boost protocols including MVA vector have also demonstrated in human clinical trials to be able to induce good T- and B-cell responses against various pathogens as, for example, human immunodeficiency virus (HIV)-1 [17,18], hepatitis C virus (HCV) [19], *Plasmodium falciparum* (the causative agent of malaria) [20], ebolavirus (EBOV) [21], and respiratory syncytial virus (RSV) [22]. Moreover, the recent development of novel vaccine candidates against COVID-19, showed heterologous prime/boost vaccination strategies able to induce more potent T-cell and humoral immune responses and higher efficacy against SARS-CoV-2 [23,24], which will help to find the best-in-class vaccine candidates.

In this study, we have analysed the ZIKV-specific T-cell and humoral immunogenicity induced in mice immunized with our previously described vaccine candidate MVA-ZIKV, in a heterologous prime/boost immunization protocol using a DNA plasmid mammalian expression vector (DNA-ZIKV) as a prime followed by MVA-ZIKV as a boost, in comparison with the corresponding homologous vectors. The results showed that the heterologous DNA-ZIKV/MVA-ZIKV immunization elicited significantly higher adaptive and memory ZIKV-specific CD4^+^ and CD8^+^ T-cell responses than homologous DNA-ZIKV/DNA-ZIKV or MVA-ZIKV/MVA-ZIKV prime/boost immunization regimens. Moreover, priming with DNA-ZIKV, also increased the magnitude of ZIKV-specific CD4^+^ T follicular helper (Tfh) cells. Furthermore, DNA-ZIKV/MVA-ZIKV also elicited a trend to higher levels of neutralizing antibodies against ZIKV than the homologous immunization regimens. Our findings reveal that the combination of DNA-ZIKV with MVA-ZIKV is an effective approach to induce ZIKV-specific T-cell and humoral immunogenicity, forming a promising vaccine strategy against ZIKV.

## Materials and methods

**Ethics statement.** The immunogenicity animal studies were approved by the Ethical Committee of Animal Experimentation (CEEA) of Centro Nacional de Biotecnología (CNB, Madrid, Spain) and by the Division of Animal Protection of the Comunidad de Madrid (PROEX 331/14) and were conducted at the CNB in a pathogen-free barrier area. Animal procedures were conformed to international guidelines and to the Spanish law under the Royal Decree (RD 53/2013).

**Cells.** Human embryonic kidney 293T (HEK293T) were grown in Dulbecco’s modified Eagle’s medium (DMEM) and 10% heat-inactivated fetal calf serum (FCS) (Gibco-Life Technologies). Vero cells (a kidney epithelial cell line from African green monkey; ATCC CCL-81) were grown in Eagle’s minimal essential medium (EMEM) and 5% heat-inactivated fetal bovine serum (Linus). Cell cultures were kept at 37°C and 5% CO_2_ in a humidified incubator.

**Viruses.** The poxvirus strains used in this study included the attenuated wild-type (WT) MVA (MVA-WT) obtained from the vaccinia virus (VACV) Ankara strain after 586 serial passages in CEF cells (derived from clone F6 at passage 585, kindly provided by G. Sutter) and the recombinant MVA-ZIKV encoding for the ZIKV prM-E structural genes (isolate Suriname Z1106033), which are inserted into the VACV TK locus of the MVA-WT genome under the transcriptional control of a novel optimized synthetic Late/Early (pLEO160) promoter [12]. Viruses grown in primary CEF cells were purified by centrifugation through two 36% (wt/vol) sucrose cushions in 10 mM Tris-HCl pH 9. All viruses were free of contamination with mycoplasma (checked by specific polymerase chain reaction (PCR) for mycoplasma), bacteria (checked by growth in LB plates without ampicillin) or fungi (checked by growth in Columbia blood agar plates; Oxoid).

ZIKV PA259459, isolated from an infected human in Panama in 2015, was propagated and titrated in semisolid agarose medium using Vero cells, as previously described [25].

**DNA vectors.** For the construction of the pCIneo-ZIKV vector (also termed DNA-ZIKV), the pCyA-ZIKV plasmid, previously used for the construction of MVA-ZIKV [12], was used as donor of the ZIKV prM-E cassette (2077bp). The ZIKV prM-E cassette was digested with *NheI* and *NotI* restriction enzymes, and then inserted into the *NheI*/*NotI*-digested pCIneo-ϕ plasmid (a mammalian cell expression vector with the CMV promoter and a neomycin-resistance marker; Promega) to generate pCIneo-ZIKV (7515 bp). The empty plasmid pCIneo-ϕ and pCIneo-ZIKV were used for prime vaccination in heterologous prime-boost protocols. *Escherichia coli* DH5α strain bacterial cultures transformed with the pCIneo-ZIKV vector were cultured in LB (Luria–Bertani) liquid medium, in the presence of ampicillin (Sigma-Aldrich, St. Louis, MO, USA) for 24 h, and then purified using an EndoFree Plasmid Mega kit (Qiagen, Hilden, Germany). The correct insertion of the ZIKV prM-E cassette was checked by PCR and DNA sequencing, as previously described [12].

**Expression of ZIKV proteins by DNA-ZIKV by Western blot.** To verify the correct expression of ZIKV prM-E proteins by the pCIneo-ZIKV (DNA-ZIKV) vector, HEK293T cells were mock transfected or transfected with 5 μg of pCIneo-ZIKV or 5 μg of empty pCIneo-ϕ vectors using PEImax (Polysciences, Warrington, PA, USA), according to the manufacturer’s recommendations. At 48 h post-transfection, cell lysates were harvested, pelleted, and resuspended in 1X Laemmli buffer plus β-mercaptoethanol. Next, cell extracts were fractionated in 10% polyacrylamide gels, and the expression of the ZIKV E protein was analysed by Western blotting using a mouse monoclonal antibody against ZIKV E (BioFront Tech; diluted 1: 5,000). Anti-rabbit horseradish peroxidase (HRP)-conjugated antibody (Sigma; diluted 1:5,000) was used as secondary antibody. The immune complexes were detected with an HRP-luminol enhanced-chemiluminescence system (ECL Plus, GE Healthcare).

**Stably cell line generation and characterization**. HeLa cells were transfected with 1 µg of pCIneo-ZIKV (DNA-ZIKV) using DharmaFECT (GE Healthcare Dharmacon) following the recommendations of the manufacturer. Selection medium supplemented with 500 μg/ml of Geneticin G-418 sulphate (Gibco) was added 24 h post-transfection. Cells were cultured in selection medium during 3 weeks and stable cell clones were obtained by limiting dilution and grown in culture medium supplemented with 500 μg/ml G-418. The expression of ZIKV E was confirmed by flow cytometry. For single-colour staining, non-transfected and transfected cells were harvested using trypsin, washed with PBS, and fixed with 4% paraformaldehyde in PBS for 15 min at RT. Fixed cells were washed with PBS and permeabilized (1% bovine serum albumin [BSA], 0.1% TritonX-100, 1M glycine in PBS) for 15 min at RT. Cells were then incubated with using mouse monoclonal anti-flavivirus antibody 4G2 (MAB10216; EMD Millipore corp.) diluted in FACS buffer (0.1% BSA, 0.01% sodium azide in PBS) for 30 min at RT. After washed in FACS buffer, cells were incubated with goat anti-mouse IgG coupled to Alexa Fluor 488 (Life Technologies) for 30 min at RT. Subsequently, cells were washed prior to analysis by flow cytometry, using a FACSCanto II cytometer (Becton Dickinson, San Jose, CA, USA). Sample analysis was performed with FlowJo 10.7.1.

**Immunofluorescence and confocal microscopy.** Procedures for immunofluorescence and confocal microscopy have been previously described [26]. Endoplasmic reticulum was labelled using rabbit anti-calnexin-CT (StressMarq Biosciences Inc.) and ZIKV E was detected using 4G2 antibody. Goat anti-rabbit IgG labelled with Alexa Fluor 594 and goat anti-mouse labelled with Alexa Fluor 488 were used as secondary antibodies.

**Enzyme-linked immunodot assay**. Cell culture medium was adsorbed to a nitrocellulose membrane by vacuum using a Bio-Dot apparatus (Bio-Rad). Membrane was blocked with 3% skimmed milk in PBS and incubated with antibody 4G2 diluted in 1% skimmed milk in PBS. After three washes with PBS the membrane was incubated with anti-mouse IgG antibody coupled to horseradish peroxidase, washed and proteins were detected by chemiluminiscence using a ChemiDocTM XRS+ System (Bio-Rad, Hercules, CA).

**Purification of VLPs by sucrose density gradient centrifugation.** Cell culture supernatants from HeLa cells stably transfected with DNA-ZIKV were harvested and VLPs were purified as previously described [27,28].

**Mouse immunization schedule.** Female BALB/c mice (6–8 weeks old) were purchased from Envigo Laboratories and stored in a pathogen-free barrier area of the CNB in accordance to the recommendations of the Federation of European Laboratory Animal Science Associations. Five groups of animals were homologous or heterologous vaccinated as follows: MVA-ZIKV/MVA-ZIKV, DNA-ZIKV/DNA-ZIKV, DNA-ZIKV/MVA-ZIKV, MVA-WT/MVA-WT and DNA-ϕ/DNA-ϕ. Animals (*n* = 8/group) received 100 μg of DNA vector (pCIneo-ZIKV or pCIneo-ϕ in the control group) or 2 × 10^7^ PFU of the corresponding MVA virus (MVA-ZIKV, or MVA-WT for control group) by bilateral intramuscular (i.m.) route (50 μl/leg: 50 μg of DNA or 1 × 10^7^ PFU of MVA per leg). Two weeks later animals were boosted by the same i.m. route with 100 μg of the corresponding DNA vectors or 2 × 10^7^ PFU of the corresponding MVA virus. At 10 and 52 days after the last immunization, 4 mice in each group were sacrificed with carbon dioxide (CO_2_). Their spleens and popliteal lymph nodes were processed to measure cellular immune responses to ZIKV antigens by intracellular cytokine staining (ICS) assay and their sera harvested and used to analyse humoral immune responses.

**Proteins and peptides.** A ZIKV E peptide pool of the ZIKV PRVABC59 strain (GenPept: AMZ03556) was used in the ICS assay for the analysis of the adaptive and memory T cell responses. Each purified peptide of the ZIKV E peptide pool is at 1 mg per vial, and was obtained through BEI Resources (National Institute of Allergy and Infectious Disease, National Institutes of Health, USA). They spanned the entire ZIKV E protein as consecutive 15-mers overlapping by 12 amino acids. Furthermore, a recombinant ZIKV E protein (Fitzgerald Industries International, Acton, MA, USA) was use as stimulus, in combination with ZIKV E peptide pool, for the analysis of Tfh response.

**Analysis of the ZIKV-specific cellular immune responses by ICS assay.**

1. **Analysis of CD4^+^ and CD8^+^ T cell responses.** The magnitude and polyfunctionality of the ZIKV-specific T cell adaptive and memory responses were analysed by ICS as previously described [12,29–31], with some modifications. After spleen processing, fresh 4 × 10^6^ splenocytes (depleted of red blood cells) were seeded onto M96 plates and stimulated for 6 h in complete RPMI 1640 medium supplemented with 10% FCS containing 1 μl/ml Golgiplug (BD Biosciences) to inhibit cytokine secretion; monensin 1X (eBioscience), anti-CD107a–FITC (BD Biosciences); and the ZIKV E peptide pool (5 μg/ml). Then cells were washed, stained for the surface markers, fixed, permeabilized (Cytofix/Cytoperm kit; BD Biosciences), and stained intracellularly with the appropriate fluorochromes. Dead cells were excluded with the violet LIVE/DEAD stain kit (Invitrogen). The fluorochrome-conjugated antibodies used for functional analyses were CD3-phycoerythrin (PE)-CF594, CD4-allophycocyanin (APC)-Cy7, CD8-V500, IFN-γ–PE-Cy7, TNF-α–PE, and IL-2–APC. In addition, the antibodies used for phenotypic analyses were CD62L-Alexa 700 and CD127-peridinin chlorophyll protein (PerCP)-Cy5.5. All antibodies were from BD Biosciences.
2. **Analysis of Tfh cell responses.** The magnitude of the ZIKV-specific Tfh cell adaptive immune responses was analysed by ICS as previously described [32–34], with some modifications. After spleen processing, fresh, 4 × 10^6^ draining lymph nodes cells (depleted of red blood cells) were seeded onto M96 plates using RPMI-10% FCS and stimulated with 5 µg/mL of ZIKV E peptide pool and 0.5 µg/mL of ZIKV E protein along with anti-CD154 (CD40L)-PE antibody at 37 °C. Two hours later, 1 µL/mL protein transport inhibitor GolgiPlug (BFA, BD Biosciences, Franklin Lakes, NJ, USA), and monensin (1X; eBioscience, Thermo Fisher Scientific, Waltham, MA, USA), were added and cells were keep incubated for 4 additional hours at 37 °C. Next, live cells were stained using fixable viability stain (FVS) 520 (BD Biosciences, Franklin Lakes, NJ, USA) for 20 min at 4°C. Then, after being washed twice with IB buffer (PBS 1X-FCS 2%-EDTA 2 mM), cells were stained for the surface markers using 50 μL of the corresponding antibodies CD4-Alexa 700, CD44-PECy5, CXCR5-PE-CF594, PD1(CD279)-APC-eFluor780 and CD8-V500 diluted following manufacturer’s instructions for 20 min at 4°C. After being washed again two times with IB buffer, splenocytes were fixed and permeabilized with BD Cytofix/Cytoperm™ solution Kit (BD Biosciences, Franklin Lakes, N.J., USA) for 20 min at 4 °C and rested overnight in IB buffer. The day after, cells were washed with Permwash 1X (BD Biosciences, Franklin Lakes, NJ, USA) and the Fc receptors were blocked with 25 μL of an anti CD16/CD32 (FcBlock) antibody (diluted 1:100 in Permwash 1X) for 5 min at 4°C. Finally, the cells were stained intracellularly for cytokines using 25 μL of intracellular antibodies IL-4-FITC, IFNγ-PECy7, and IL-21-APC (diluted following manufacturer’s instructions) for 20 min at 4°C and washed then twice in Permwash 1X after resuspended them in 200 μL of IB buffer.

**Plaque reduction neutralization assay (PRNT).** Titers of neutralizing antibodies against ZIKV present in the sera of immunize mice were determined by a PRNT assay using Vero cells, as previously described [27]. Titers of neutralizing antibodies were expressed as the reciprocal of the serum dilution that inhibited plaque formation by 50% (PRNT50), relative to samples incubated with negative control sera.

**Statistical procedures.** Statistical analysis of the ICS assay results was realized as previously described [35], by an approach that corrects measurements for the medium response (RPMI), calculating confidence intervals and *P* values. Only antigen response values significantly larger than the corresponding RPMI are presented. Background values were subtracted from all of the values used to allow analysis of proportionate representation of responses. The statistical significance of neutralization measurement (PRNT50) in Balb/c sera was determined by an unpaired t-test. The statistical significances are indicated as follows: *, *P* < 0.05; **, *P* < 0.005; ***, *P* < 0.001.

## Results

**Figure 1.**
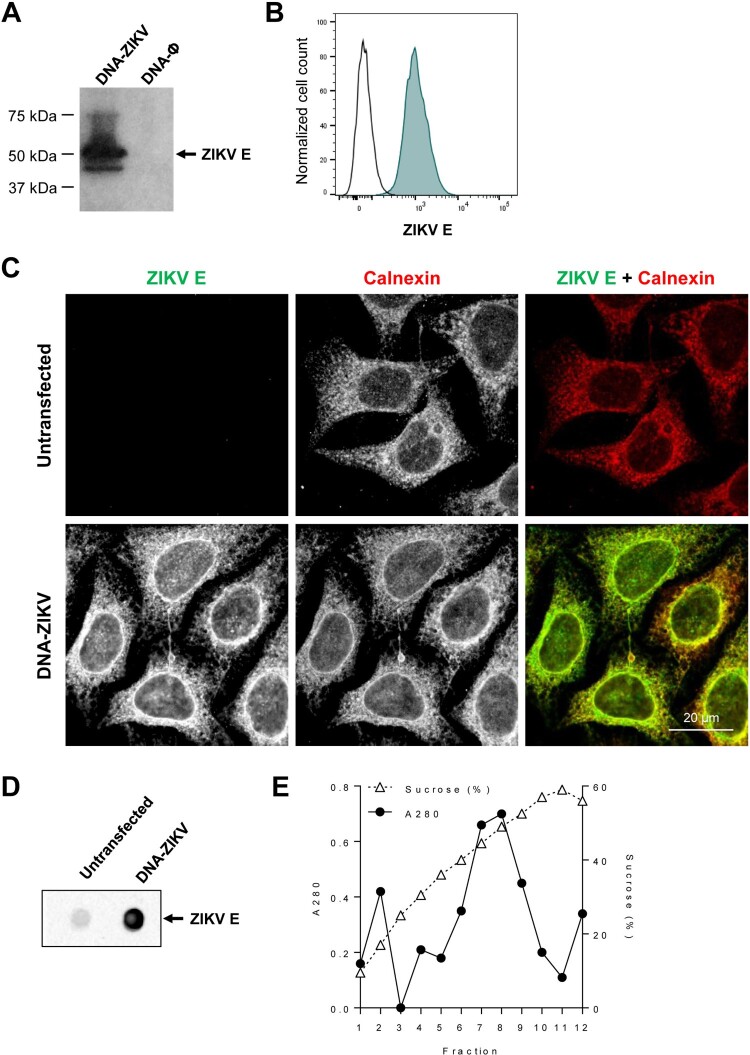
DNA-ZIKV vector express ZIKV E protein. (A) HEK-293T cells were transfected with 5µg of pCIneo-ZIKV (DNA-ZIKV) or empty pCIneo-ϕ vectors. At 48 h post-transfection, cell extracts were analysed by Western blot against ZIKV E. Arrow on the right indicate the position of the ZIKV E protein. The sizes of standards (in kDa) are indicated on the left. (B) Expression of ZIKV E from a stable cell line of transfected HeLa cells. Transfected or untransfected control cells were fixed, permeabilized and ZIKV E protein was detected using 4G2 antibody and Alexa Fluor 488 secondary antibodies. The expression of ZIKV E in the stable HeLa cell clone was analysed by flow cytometry. (C) Immunofluorescence analysis of ZIKV E expression in a cell clone of HeLa cells transfected with DNA-ZIKV. Zika virus E (green) was detected as described in (B) and the endoplasmic reticulum was detected using rabbit anti-Calnexin antibodies and Alexa Fluor-594 (red) secondary antibodies. Scale bar: 20 µm. (D) Enzyme-linked immunodot assay using 4G2 monoclonal antibody of culture supernatants from a HeLa cell clone transfected with DNA-ZIKV. Culture medium from untransfected HeLa cells was included as a negative control. (E) Purification of ZIKV VLPs from the supernatant of a HeLa cell clone transfected with DNA-ZIKV by sucrose density gradient ultracentrifugation. A280 denotes the amount of protein determined by spectrophotometry (absorbance at 280 nm) in fractions obtained after ultracentrifugation of concentrated supernatants loaded into a 20–60% w/v sucrose gradient. Sucrose density was also determined by refractometry of each fraction.

**Generation of a DNA-based vaccine vector expressing prM-E ZIKV antigens (DNA-ZIKV).** We developed a DNA-based mammalian expression vector expressing ZIKV prM-E proteins, termed DNA-ZIKV, as described in Materials and Methods. The correct expression of the ZIKV E protein from the recombinant DNA-ZIKV vector was confirmed and evaluated from cell extracts of transiently transfected HEK293T cells by Western blot (Figure 1(A)). In addition, by flow cytometry high levels of E protein were observed in a cell clone of stable DNA-ZIKV transfected HeLa cells (Figure 1(B)). Immunofluorescence analysis of stable transfected cells showed that, as expected, ZIKV E glycoprotein co-localized with calnexin, an endoplasmic reticulum marker (Figure 1(C)). Following synthesis in the endoplasmic reticulum, flavivirus prM and E assemble into virus-like particles (VLPs) that enter into the secretory pathway prior to be released into the extracellular medium [36]. Accordingly, the analysis of the cell culture medium by dot blot confirmed that the expression of DNA-ZIKV induced the release of ZIKV antigens to the extracellular medium (Figure 1(D)). Sucrose gradient centrifugation analysis supported the particulate nature of the released antigens that was compatible with the production of VLPs (Figure 1(E)).

**Figure 2.**
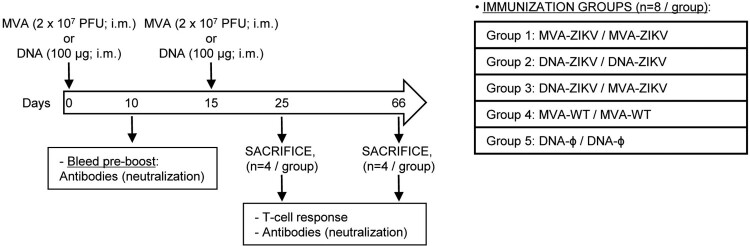
Immunization schedule. Five groups of female BALB/c mice (6–8 weeks old) were homologous or heterologous vaccinated as follows: MVA-ZIKV/MVA-ZIKV, DNA-ZIKV/DNA-ZIKV, DNA-ZIKV/MVA-ZIKV, MVA-WT/MVA-WT and DNA-ϕ/DNA-ϕ. Animals (*n* = 8/group) received 100 μg of DNA vector (pCIneo-ZIKV or pCIneo-ϕ in the control group) or 2 × 10^7^ PFU of the corresponding MVA virus (MVA-ZIKV, or MVA-WT for control group) by bilateral intramuscular (i.m.) route. Two weeks later animals were boosted by the same i.m. route with 100 μg of the corresponding DNA vectors or 2 × 10^7^ PFU of the corresponding MVA virus. At 10 and 53 days after the last immunization, 4 mice in each group were sacrificed with carbon dioxide (CO_2_). Their spleens and popliteal lymph nodes were processed to measure cellular immune responses to ZIKV antigens by intracellular cytokine staining (ICS) assay and their sera harvested and used to analyse humoral immune responses.

**Heterologous prime/boost immunization in mice with DNA-ZIKV followed by MVA-ZIKV increases the magnitude of adaptive ZIKV-specific CD4^+^ and CD8^+^ T cell immune responses.** Cellular immune responses are pivotal for protection against ZIKV infection [37,38]. Thus, the ZIKV-specific immunogenicity elicited in mice by DNA-ZIKV or MVA-ZIKV vaccine candidates expressing ZIKV prM-E proteins was examined following homologous or heterologous prime/boost immunizations. Thus, Balb/c mice (*n* = 8/group) were immunized as shown in Figure 2 using the following groups: MVA-ZIKV/MVA-ZIKV, DNA-ZIKV/DNA-ZIKV, DNA-ZIKV/MVA-ZIKV, MVA-WT/MVA-WT and DNA-ϕ/ DNA-ϕ, and as described in Materials and Methods. At 10 days post-boost half of the animals were sacrificed and adaptive ZIKV-specific T cell (CD4, CD8 and Tfh) immune responses were analysed by a polychromatic intracellular cytokine staining (ICS) assay. To detect the ZIKV-specific T cell responses, splenocytes from immunized animals were stimulated *ex vivo* for 6 h with ZIKV-specific peptide pools spanning the entire ZIKV E protein. The percentages of CD4^+^ and CD8^+^ T cells that produced IFN-γ and/or IL-2 and/or TNF-α and/or CD107a established the overall magnitude of ZIKV-specific CD4^+^ or CD8^+^ T cell immune responses. Mice immunized with two doses sham DNA (DNA-ϕ) or non-recombinant MVA-WT were used as controls.

**Figure 3.**
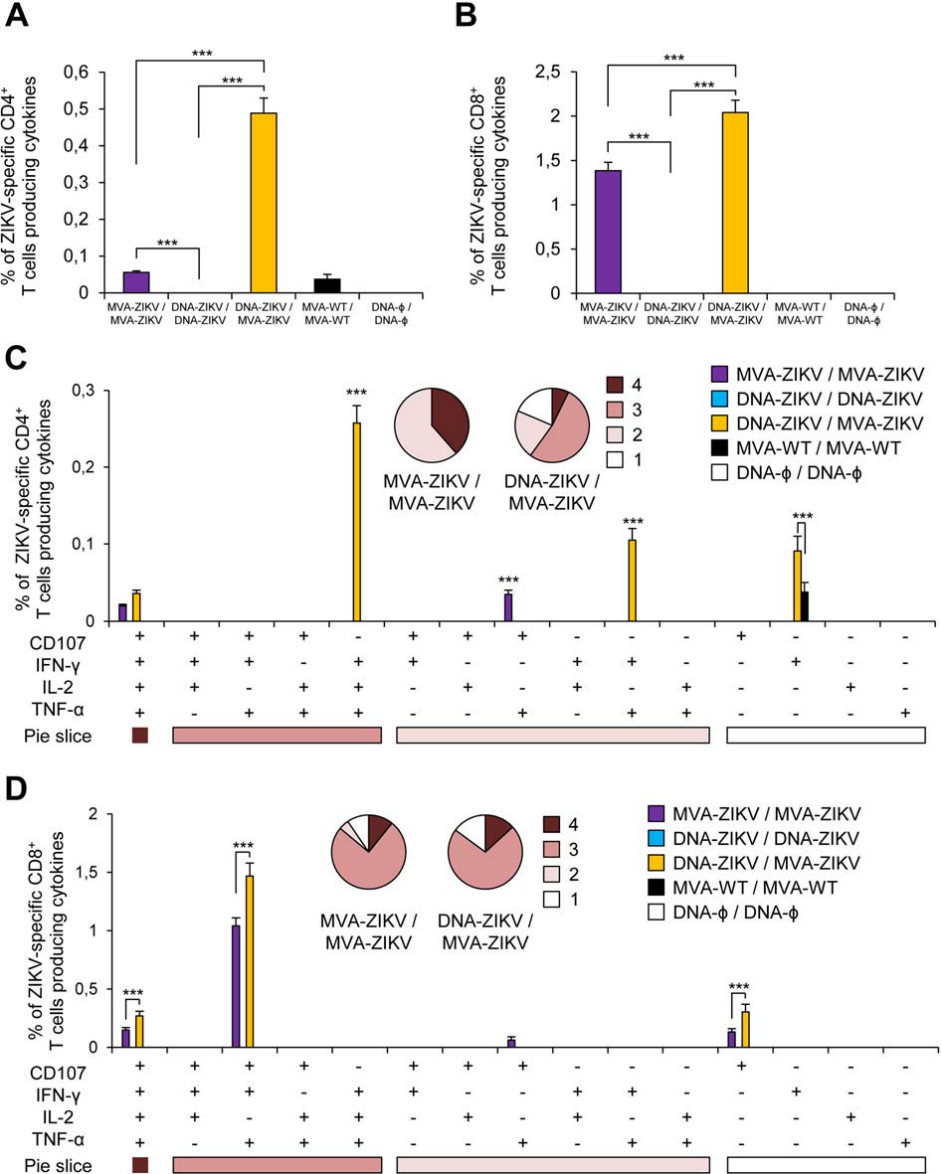
Adaptive ZIKV-specific CD4^+^ and CD8^+^ T cell immune responses in immunized mice. Splenocytes were collected from mice (*n* = 4 per group) immunized with MVA-ZIKV/MVA-ZIKV, DNA-ZIKV/DNA-ZIKV, DNA-ZIKV/MVA-ZIKV, MVA-WT/MVA-WT and DNA-ϕ/ DNA-ϕ, 10 days after the last immunization. Next, ZIKV-specific CD4^+^ and CD8^+^ T cell adaptive immune responses triggered by the different immunization groups were measured by ICS assay following the stimulation of splenocytes with an ZIKV E peptide pool. Values from unstimulated controls were subtracted in all cases. (A and B) Magnitude of the total ZIKV-specific CD4^+^ (A) and CD8^+^ (B) T cell responses after stimulation of splenocytes with the ZIKV E peptide pool. The total value in each group represents the sum of the percentages of CD4^+^ and CD8^+^ T cells expressing CD107a and/or producing IFN-γ and/or IL-2 and/or TNF-α against ZIKV E peptide pool. (C and D) Polyfunctionality of the ZIKV-specific CD4^+^ (C) and CD8^+^ (D) T cell responses shown as the combined production of CD107a and/or IFN-γ and/or IL-2 and/or TNF-α against the ZIKV E peptide pool. *p* values indicate significant response differences between immunization groups (*** *p* < 0.001). Responses are grouped and colour coded on the basis of the number of functions (4, 3, 2, or 1). The pie charts summarize the data. Each slice corresponds to the proportion of the total ZIKV-specific CD4^+^ and CD8^+^ T cells exhibiting 1, 2, 3, or 4 functions (CD107a and/or IFN-γ and/or TNF-α and/or IL-2).

The results showed that homologous MVA-ZIKV/MVA-ZIKV and heterologous DNA-ZIKV/MVA-ZIKV prime/boost approaches elicited good levels of ZIKV-specific CD4^+^ and CD8^+^ T cell immune responses, which were mediated largely by the CD8^+^ T cell subset (Figure 3(A,B)). However, no ZIKV-specific CD4^+^ or CD8^+^ T cell responses were detected after homologous DNA-ZIKV/DNA-ZIKV immunization. The comparison of the CD4^+^ and CD8^+^ T cell responses induced by heterologous DNA-ZIKV/MVA-ZIKV and homologous MVA-ZIKV/MVA-ZIKV showed that DNA-ZIKV/MVA-ZIKV elicited a significant higher magnitude of ZIKV-specific CD4^+^ and CD8^+^ T cell responses (around 8-fold and 1.4-fold higher) compared with the homologous regimen (*p* < 0.001) (Figure 3(A,B)).

The quality of a T cell response can be characterized by the profile of cytokine production and cytotoxic potential. Thus, based on the analysis of IFN-γ, IL-2, and TNF-α secretion and surface mobilization of CD107a on activated T cells as an indirect marker of cytotoxicity, 15 different ZIKV-specific CD4^+^ and CD8^+^ T cell populations could be identified (Figure 3(C,D)). CD4^+^ T cells expressing IFN-γ+TNF-α+IL-2, IFN-γ+ TNF-α, and IFN-γ were the most induced populations elicited by heterologous DNA-ZIKV/MVA-ZIKV immunization group, whereas CD4^+^ T cells expressing CD107a + TNF-α, and CD107a + IFN-γ+TNF-α+IL-2 were the most induced populations elicited by homologous MVA-ZIKV/MVA-ZIKV immunization group (Figure 3(C)). DNA-ZIKV/MVA-ZIKV elicited a significant higher magnitude of those ZIKV-specific CD4^+^ T cell populations, with more than 60% of the CD4^+^ T cells exhibiting three or four more functions (Figure 3(C), pie charts).

On the other hand, CD8^+^ T cells expressing CD107a + IFN-γ+TNF-α, CD107a + IFN-γ+TNF-α+IL-2, and CD107a were the most induced populations elicited by both heterologous DNA-ZIKV/MVA-ZIKV and homologous MVA-ZIKV/MVA-ZIKV immunization groups. Heterologous DNA-ZIKV/MVA-ZIKV immunization group induced a significantly greater percentage of the major populations than MVA-ZIKV/MVA-ZIKV immunization group (Figure 3(D)), but both immunization groups had a similar polyfunctionality profile of ZIKV-specific CD8^+^ T-cell responses, with 90% (MVA-ZIKV/MVA-ZIKV) and 85% (DNA-ZIKV/MVA-ZIKV) of the CD8^+^ T cells exhibiting two or more functions (Figure 3(D), pie charts).

**Heterologous prime/boost immunization in mice with DNA-ZIKV followed by MVA-ZIKV enhances the levels of ZIKV-specific CD4^+^ T follicular helper (Tfh) cell responses.** CD4^+^ T follicular helper (Tfh) cells are a subpopulation of T helper cells involved in the development and sustaining of germinal centre (GC) interactions, an essential crosstalk that promotes the generation of long-lived high-affinity humoral immunity [39]. Since the interaction between Tfh and B cells is mediated both by cell-associated and soluble factors, including CD154 (CD40L), IFN-γ, and IL-4 [40], the ZIKV-specific CD4^+^ Tfh cell responses were studied analysing those parameters by ICS assay in draining (popliteal) lymph nodes (DLN) obtained from immunized mice at 10 days after the last immunization. Thus, DLN cells were non-stimulated (RPMI) or stimulated *ex vivo* for 6 h with ZIKV E protein plus ZIKV peptide pool. Double positive CXCR5^+^/PD-1^+^ cell population gated on CD4^+^ T cells defined total Tfh cells, whereas percentages of Tfh cells that produced IL-4 and/or IFN-γ and/or expressed CD154 (CD40L) established the ZIKV-specific Tfh responses.

**Figure 4.**
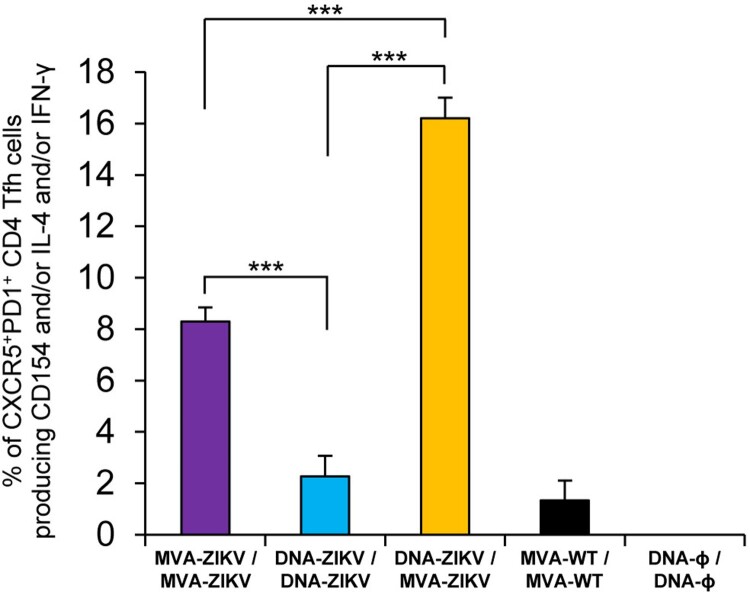
ZIKV-specific Tfh cell immune responses. Mice (*n* = 4) were immunized with MVA-ZIKV/MVA-ZIKV, DNA-ZIKV/DNA-ZIKV, DNA-ZIKV/MVA-ZIKV, MVA-WT/MVA-WT and DNA-ϕ/ DNA-ϕ. At 10 days after the last immunization, the magnitude of ZIKV-specific CD4^+^ Tfh cell immune response was studied in popliteal (draining) lymph nodes by ICS assay. The total value in each group represents the sum of the percentages of CD4^+^ Tfh cells expressing CD154 and/or producing IL-4 and/or IFN-γ against ZIKV E protein plus ZIKV E peptide pool. Data are background (RPMI)-subtracted. *P* values indicate significant response differences between immunization groups (***, *p* < 0.001).

The results showed that the magnitude of ZIKV-specific Tfh response was significantly higher in DLN of animals immunized with heterologous DNA-ZIKV/MVA-ZIKV than in those immunized with homologous MVA-ZIKV/MVA-ZIKV or DNA-ZIKV/DNA-ZIKV (Figure 4). Moreover, the magnitude of ZIKV-specific Tfh response in DLN of animals immunized with homologous MVA-ZIKV/MVA-ZIKV was higher than in those immunized with homologous DNA-ZIKV/DNA-ZIKV (Figure 4).

**Heterologous DNA-ZIKV/MVA-ZIKV prime/boost immunization maintains higher magnitude of memory ZIKV-specific CD4^+^ and CD8^+^ T cell immune responses than the homologous vector combinations.** Memory cellular immune responses are also important for protection against ZIKV infection [38]. Thus, next we analysed at 53 days post-boost the ZIKV-specific CD4^+^ and CD8^+^ T cell memory immune responses induced in mice by the different immunization groups.

**Figure 5.**
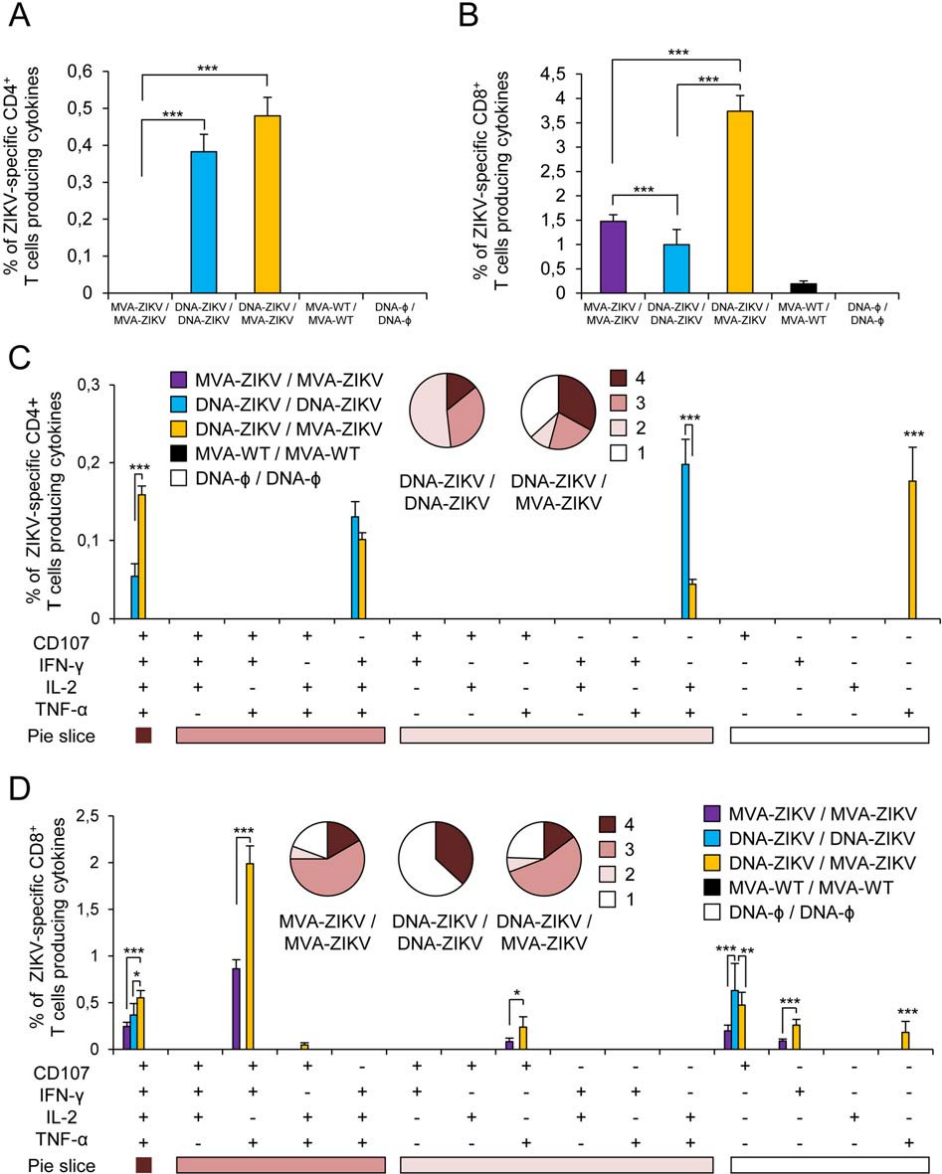
Memory ZIKV-specific CD4^+^ and CD8^+^ T cell immune responses in immunized mice. Splenocytes were collected from mice (*n* = 4 per group) immunized with MVA-ZIKV/MVA-ZIKV, DNA-ZIKV/DNA-ZIKV, DNA-ZIKV/MVA-ZIKV, MVA-WT/MVA-WT and DNA-ϕ/ DNA-ϕ, 53 days after the last immunization. Next, ZIKV-specific CD4^+^ and CD8^+^ T cell memory immune responses triggered by the different immunization groups were measured by ICS assay following the stimulation of splenocytes with a ZIKV E peptide pool. Values from unstimulated controls were subtracted in all cases. (A and B) Magnitude of the total ZIKV-specific CD4^+^ (A) and CD8^+^ (B) T cell responses after stimulation of splenocytes with the ZIKV E peptide pool. The total value in each group represents the sum of the percentages of CD4^+^ and CD8^+^ T cells expressing CD107a and/or secreting IFN-γ and/or IL-2 and/or TNF-α against ZIKV E peptide pool. (C and D) Polyfunctionality of the ZIKV-specific CD4^+^ (C) and CD8^+^ (D) T cell responses shown as the combined production of CD107a and/or IFN-γ and/or IL-2 and/or TNF-α against the ZIKV E peptide pool. *p* values indicate significant response differences between immunization groups (*** *p* < 0.001). Responses are grouped and colour coded on the basis of the number of functions (4, 3, 2, or 1). The pie charts summarize the data. Each slice corresponds to the proportion of the total ZIKV-specific CD4^+^ and CD8^+^ T cells exhibiting 1, 2, 3, or 4 functions (CD107a and/or IFN-γ and/or TNF-α and/or IL-2).

Again, the heterologous vector combination elicited the highest levels of ZIKV-specific CD4^+^ and CD8^+^ T cell immune responses, and triggered an overall ZIKV-specific immune response mediated mainly by CD8^+^ T cells (Figure 5(A,B)). Surprisingly, no apparent ZIKV-specific CD4^+^ T cell memory immune responses were detected at this timepoint in animals immunized with MVA-ZIKV/MVA-ZIKV. Similarly, while in the group of DNA-ZIKV/DNA-ZIKV there was no apparent adaptive CD4 and CD8+ T cell responses at 10 days post boost (see Figure 3(A,B)), however memory responses were well observed at 53 days (Figure 5(A,B)). The comparison of the CD4^+^ and CD8^+^ T cell memory responses induced by all the immunization groups showed that, as in the adaptive phase, heterologous DNA-ZIKV/MVA-ZIKV was the most immunogenic group, and elicited a significant higher magnitude of ZIKV-specific CD4^+^ T cell memory responses than DNA-ZIKV/DNA-ZIKV (around 1-fold higher) (Figure 5(A)) and a significant higher magnitude of ZIKV-specific CD8^+^ T cell memory responses than DNA-ZIKV/DNA-ZIKV or MVA-ZIKV/MVA-ZIKV (around 2.5-fold and 3.5-fold higher, respectively) (*p* < 0.001) (Figure 5(B)).

Analysis of the quality of ZIKV-specific CD4^+^ and CD8^+^ T cell memory immune responses showed that CD4^+^ T cells expressing TNF-α, CD107a + IFN-γ+TNF-α+IL-2, IFN-γ+TNF-α+IL-2, and TNF-α+IL-2 were the most induced populations elicited by heterologous DNA-ZIKV/MVA-ZIKV immunization group, whereas CD4^+^ T cells expressing TNF-α+IL-2, IFN-γ+TNF-α+IL-2 and CD107a + IFN-γ+TNF-α+IL-2 were the most induced populations elicited by homologous DNA-ZIKV/DNA-ZIKV immunization group (Figure 5(C)). In both immunization groups more than 50-55% of memory ZIKV-specific CD4^+^ T cells exhibited three or four functions (Figure 5(C), pie charts). On the other hand, CD8^+^ T cells expressing CD107a + IFN-γ+TNF-α and CD107a + IFN-γ+TNF-α+IL-2 were the most induced populations elicited by heterologous DNA-ZIKV/MVA-ZIKV and homologous MVA-ZIKV/MVA-ZIKV immunization groups (Figure 3(D)). Again, MVA-ZIKV/MVA-ZIKV and DNA-ZIKV/MVA-ZIKV immunization groups exhibited similar polyfunctionality profiles, with 76% and 81% of memory ZIKV-specific CD8^+^ T cells exhibiting two or more functions, but in the DNA-ZIKV/DNA-ZIKV immunization group 63% of memory ZIKV-specific CD8^+^ T cells present one single function (Figure 5(D), pie charts). In both cases (CD4^+^ and CD8^+^ T cells), heterologous DNA-ZIKV/MVA-ZIKV immunization group induced a significantly greater percentage of most of the major populations than the homologous immunization groups (Figure 5(C,D)).

**DNA-ZIKV/MVA-ZIKV immunization induces neutralizing antibodies against ZIKV in mice.** Neutralizing antibodies against ZIKV are critical to control ZIKV infection [41,42]. Thus, to evaluate the capability of the different immunization groups to induce neutralizing antibodies against ZIKV, we determined the plaque reduction neutralization titters that neutralized 50% of ZIKV (PRNT50) in serum samples obtained from immunized Balb/c mice at 10 and 53 days after the last immunization.

**Figure 6.**
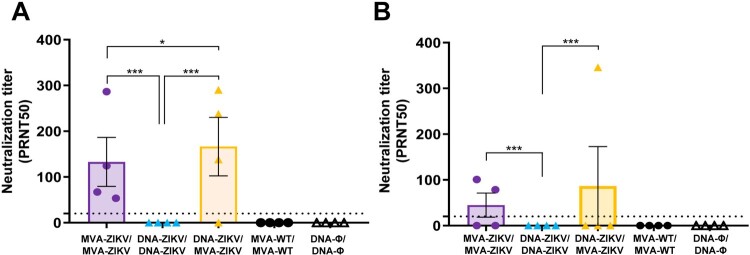
Induction of neutralizing antibodies by MVA-ZIKV in immunocompetent mice. Mice (*n* = 4) were immunized with MVA-ZIKV/MVA-ZIKV, DNA-ZIKV/DNA-ZIKV, DNA-ZIKV/MVA-ZIKV, MVA-WT/MVA-WT and DNA-ϕ/ DNA-ϕ and 10 (A) and 53 (B) days after the last immunization ZIKV-neutralizing antibody titters were analysed. Data represent the reciprocal of the serum dilution that inhibited plaque formation by 50% (PRNT50), relative to samples incubated with negative control sera. Dashed line indicates the limit of detection (LOD) of the neutralization assay (1/20 dilution).

The results showed that individual serum samples obtained from mice immunized with DNA-ZIKV/MVA-ZIKV and MVA-ZIKV/MVA-ZIKV neutralized ZIKV (PA259459 strain, from Asian-American lineage) (Figure 6), with DNA-ZIKV/MVA-ZIKV eliciting a trend of greater mean-neutralization titter (166) than MVA-ZIKV/MVA-ZIKV (133) in the adaptive phase, at 10 days post-boost (Figure 6(A)). Furthermore, at the memory phase (53 days post-boost), the same pattern is observed, but with lower PRNT50 neutralization titters (Figure 6(B)). However, we could not detect neutralizing antibodies against ZIKV in serum samples obtained from mice immunized with the DNA-ZIKV/DNA-ZIKV protocol.

## Discussion

ZIKV is an important emerging flavivirus transmitted by infected mosquitoes from the genus *Aedes* that can cause severe complications in humans [1]. The virus has caused recent outbreaks of the disease worldwide and their future expansion to novel geographical areas is highly possible [4]. Several different ZIKV vaccine candidates under different platforms have now been developed and tested in preclinical and clinical trials, but none of them have been licensed yet. These include: nucleic acid vaccines (DNA and RNA vaccines), inactivated whole virus vaccines, live attenuated vaccines, viral vectored vaccines, protein antigen vaccines in the form of purified proteins from expression systems, or VLPs [6].

One of the most promising vaccine platforms developed against ZIKV are the recombinant viral vectors. These include vesicular stomatitis virus (VSV), measles virus (MV), adenovirus (Ad), and vaccinia virus (VACV). An attenuated version of a recombinant VSV expressing the prM-E of ZIKV elicited both neutralizing antibody responses and T cell responses that protected challenged newborn mice born to vaccinated female mice [43]. A live attenuated measles virus vector expressing prM and soluble E (MV-Zika-sE) was shown to protect mice from ZIKV challenge through the development of E-specific neutralizing antibodies and cellular immune responses [44]. The immunization of NHPs also resulted in ZIKV-specific neutralizing antibody responses in all vaccinated animals. This vaccine and another similarly developed MV vaccine (MV-ZIKV-RSP) are in human phase I clinical trials [6]. Furthermore, several adenovirus vectors expressing prM-E and/or E alone have been shown to induce neutralizing antibodies and cellular immune responses that confer protection in mice and NHPs [5,41]. There are also several VACV recombinant vectors as ZIKV vaccine candidates. A two-dose regimen using DNA and/or non-replicating VACV-based (Tian Tan strain) vaccine candidates expressing ZIKV prM-E cassette elicited in Balb/c mice robust neutralizing activity against ZIKV both in a homologous DNA/DNA and VACV/VACV immunization protocols just as much as in a heterologous DNA/VACV immunization protocol, as well as similar production of ZIKV E-specific IgG antibodies [45]. Their results also showed that DNA-prME/VACV-prME heterologous immunization lead to a significantly greater level of T cell response than both DNA/DNA and VACV/VACV homologous immunization protocols [45]. Various MVA recombinant viruses constructed to express the ZIKV E protein with modifications on the precursor membrane (prM) protein or on the C-terminus envelope transmembrane domain (TM) were evaluated as a non-adjuvanted single vaccination regimen against a ZIKV Brazilian isolate in BALB/c mice, demonstrating a low induction of cellular responses but good anti-ZIKV E titers that were enough immunogenic to substantially reducing the ZIKV loads in blood after challenge [46]. Another MVA-based single vector construct expressing prM-E of ZIKV and the structural proteins of chikungunya virus (C-E3-E2-6K-E1) induced neutralizing antibody responses to both viruses in immunocompetent and immunocompromised mice and blocked ZIKV viremia and disease [47]. Additionally, this vaccine candidate also blocked the vertical transmission of ZIKV in immunocompromised female mice and testes damage in male mice [47]. Other MVA-based ZIKV vaccine candidate, this time, targeting NS1 protein was able to protect immunocompetent adult mice in a lethal challenge model [48].

Thus, MVA-based vectors could be promising vaccine candidates against ZIKV. We have recently described the generation of MVA-ZIKV, an MVA-based ZIKV vaccine candidate expressing ZIKV prM-E antigens able to form ZIKV VLPs, which was highly immunogenic in mice inducing robust levels of ZIKV-specific CD4^+^ and CD8^+^ T cell immune responses, as well as high levels of neutralizing antibodies [12]. Remarkably, one single dose of MVA-ZIKV was able to control Zika virus replication in a challenged mouse model [12]. However, although the MVA-ZIKV vaccine candidate generated was highly immunogenic and protective in susceptible mice models at short timepoints, novel vaccine candidates and/or immunization approaches that could improve the magnitude and durability of the ZIKV-specific immune responses are desirable. This is particularly noticeable with the current SARS-CoV-2 pandemic where vaccines able to induce broad B and T cell responses with long-lasting immunity are sought.

Here, we reported that a priming immunization with regular mammalian expression DNA vector encoding the ZIKV prM-E antigens (DNA-ZIKV) followed by a boost with MVA-ZIKV (expressing the same prM-E antigens) induced a significant higher magnitude of ZIKV-specific CD4^+^ and CD8^+^ T cell immune responses, as well as higher levels of neutralizing antibodies, than either DNA-ZIKV/DNA-ZIKV or MVA-ZIKV/MVA-ZIKV immunizations.

Accumulating evidence suggests that both cellular and humoral responses are required for effective control of ZIKV [49,50]. Related to T cell responses, several depletion studies, in which loss of either CD4^+^, CD8^+^, or both T cell subsets together, have demonstrated a worsened morbidity, mortality, or even foetal resorption in different ZIKV infection models, suggesting an important role for CD4^+^ and CD8^+^ T cells in the immune response to ZIKV [51]. Additionally, CD4^+^ and CD8^+^ T-cell responses induced by vaccination with a DNA-based ZIKV vaccine candidate in Phase I clinical trial might have been relevant for efficacy and immunogenicity [52].

On the one hand, CD4^+^ T cells have been proved to be important mediators of protection against ZIKV, in several infection and vaccination context [37,51]. In our previous study, we were not able to detect ZIKV-specific CD4^+^ T cell responses when immunized mice with two doses of MVA-ZIKV [12]. Here, consistently with our previous results, we have barely or no observed ZIKV-specific CD4^+^ T cell responses within the limits of detection, when mice were immunized with two doses of MVA-ZIKV (in both time-points studied, 10- and 53-days post-immunization); in the case of DNA-ZIKV, there was no CD4^+^ T cell response at 10 days after the booster but good levels at 53-days post boost, indicating a delay immune response triggered by the DNA vector. However, a significant increase in the magnitude of adaptive and memory ZIKV-specific CD4^+^ T cell responses was achieved when a DNA-ZIKV/MVA-ZIKV heterologous prime/boost regimen was applied. On the other hand, recent studies have confirmed the importance of CD8^+^ T cells in ZIKV infection and in vaccine-induced protection [51,53,54]. Furthermore, in a ZIKV infection model it has been described that CD8^+^ T cells are capable of local viral control if they arrive in the brain early after viral invasion, demonstrating the benefits of considering this subset when designing vaccines against ZIKV virus [38]. Our work, demonstrates that a combined immunization with DNA-ZIKV/MVA-ZIKV was able to enhance significantly the levels of adaptive and memory ZIKV-specific CD8^+^ T cell responses expressing important cytokines as IFN-γ, TNF-α, IL-2 and the degranulation marker CD107a. A 2018 study observed that an Adenovirus-vectored vaccine containing the ZIKV prM-E elicited robust humoral and cellular immune responses (but lower than those elicited with our DNA-ZIKV/MVA-ZIKV) in immunocompetent BALB/c mice and provided sterilizing protection against ZIKV infection in Interferon receptor-deficient (A129) mice [55]. Other VSV-based vaccine candidate covering the prM-E region of ZIKV that also elicits robust CD8^+^ T cell responses, but lower than those achieved by our DNA-ZIKV/MVA-ZIKV protocol, was able to protect C57BL/6 mice from morbidity or mortality and Neonatal mice born from vaccinated mother were protected from ZIKV replication after challenge [43].

Another important achievement of this work is the study, for the first time for a ZIKV vaccine candidate, of the vaccine induced Tfh responses. Naive CD4^+^ T cells can differentiate into Th1 and Tfh cells during viral infection [39]. Th1 cells control virus spread, and provide help to the generation and maintenance of cytotoxic T lymphocytes, whereas Tfh cells are involved in the development and sustaining of germinal centre (GC) interactions and, therefore, in the induction of antibody responses. Recent studies found an indispensable role of Tfh cells for the elicitation of ZIKV-specific neutralizing antibodies and long-term maintenance of antibody response [37], pivotal for an efficacious vaccine. In concordance with this important role of Tfh on ZIKV infection, we describe here that the MVA-ZIKV vaccine candidate is able to induce potent levels of ZIKV-specific Tfh cells, either when used in a homologous MVA-ZIKV/MVA-ZIKV immunization protocol or when used in a heterologous DNA-ZIKV/MVA-ZIKV immunization regimen. Again, similarly to what we observed with ZIKV-specific CD4^+^ and CD8^+^ T cell responses, the heterologous DNA-ZIKV/MVA-ZIKV immunization protocol was the most immunogenic regimen inducing significant higher magnitudes of ZIKV-specific Tfh cells, expressing IFN-γ, IL-4 and/or CD40L than the other regimens used.

The most relevant correlate established in mice and rhesus monkeys was between the level of neutralizing antibodies induced upon ZIKV vaccination and the immune protection against ZIKV challenge [5,41]. Our previous results demonstrated that MVA-ZIKV induced neutralizing antibodies against ZIKV and was able to significantly reduce ZIKV viremia after a challenge with ZIKV in IFNAR^-/-^ mice (non-lethal challenge mouse model) [12]. In fact, the higher reduction in ZIKV viremia was observed in animals immunized with a two-dose MVA-ZIKV regimen that was the immunization protocol eliciting higher levels of neutralizing antibodies compared to a one-dose regimen, reflecting a correlation with levels of neutralizing antibodies and protection [12]. Here, we report that immunization with a heterologous DNA-ZIKV/MVA-ZIKV regimen induced a trend to higher neutralizing antibody titers against ZIKV, compared with the previously described and efficacious MVA-ZIKV/MVA-ZIKV homologous immunization protocol. A recombinant VSV-based ZIKV vaccine candidate expressing ZIKV genes for the prM and E proteins (from ZIKV strain ZikaSPH2015) was able to generate a high titer of neutralization Ab, in a similar range as our MVA-ZIKV/MVA-ZIKV and DNA-ZIKV/MVA-ZIKV immunization protocols did, and also demonstrated that progeny of vaccinated females are largely protected from lethal ZIKV infection [43]. Later in 2018, Bullard and colleagues stated that prime/boost strategies utilizing Adenovirus-based vaccines candidates expressing ZIKV prM and E proteins as the prime induced a strong anti-ZIKV humoral immune response, with neutralization titters measured as PRNT50 reached 400. Nonetheless, the study showed that vaccination strategies with these kind of recombinant vaccines that elicited lower neutralization titters, in a similar range that the ones elicited by our MVA-ZIKV/MVA-ZIKV and DNA-ZIKV/MVA-ZIKV immunization protocols, were as efficacious as higher neutralizing titters protecting anti-Ifnar1 mAb-treated C57BL/6 mice challenged with ZIKV against weight loss and death [56].

The analysis of correlates of protection in ZIKV infection is important to define what immune parameters are linked with protection, and are vital to design and produce better vaccines or immunization protocols. In general, it is considered in the vaccine field that a vaccine should trigger both B and T cell immune responses to accomplish the elimination of the pathogen, with T cells playing a more important role in the long-term efficacy. Depending on the nature of the vaccine, leading to activation of B over T cells, vice versa or more balanced, these parameters might contribute differentially to the protective responses. We have previously demonstrated full efficacy of the homologous prime/boost immunization with MVA-ZIKV, and even with a single dose of MVA-ZIKV [12]. In the current study, we could assume the same efficacy of the heterologous DNA-ZIKV/MVA-ZIKV prime/boost immunization, as we achieved better immune responses in all immune parameters analysed. Efficacy experiments to extend correlates of protection (antibodies and CD4^+^ or CD8^+^ T cells) of the DNA-ZIKV and MVA-ZIKV vaccine candidates, used in homologous or heterologous prime/boost immunization protocols, will be tested in the future.

Lastly, a ZIKV vaccine should ideally elicit long-lasting immunogenicity [57]. Thus, long-term follow-up experiments of memory T cell analysis would help to further examine the protection property of our different immunization schedules with DNA-ZIKV and MVA-ZIKV. To date, there are limited information about the possible relationship between memory T cell responses and the longevity of vaccine efficacy against ZIKV, but it is known that ZIKV can infect individuals for long periods, indicating that the generation of effective memory T lymphocytes activity may be important in helping to protect against ZIKV infection [51]. Besides, different recombinant MVA vaccine candidates have proved in clinical assays to be able to induce long-lasting immune responses [58,59]. Our results showed that immunizing with a heterologous DNA-ZIKV/MVA-ZIKV regimen increased the magnitude of the memory ZIKV-specific CD4^+^ and CD8^+^ T cell responses compared with homologous regimens MVA-ZIKV/MVA-ZIKV and DNA-ZIKV/DNA-ZIKV, which is one of the most commonly vaccination strategies in the ongoing clinical trials performed for ZIKV [52,60]. Moreover, we have described the presence of neutralizing antibodies against ZIKV at the memory phase, showing that this heterologous DNA-ZIKV/MVA-ZIKV regimen is able to induce also durable humoral immune responses. Thus, the ability of the DNA-ZIKV/MVA-ZIKV immunization regimen to elicit high memory ZIKV-specific T cell responses and neutralizing antibodies adds more value to this regimen in potentiating durable responses that might extend vaccine protection.

Overall, in this investigation we have established an immunization regimen based on DNA-ZIKV as a prime followed by MVA-ZIKV as a boost, that elicited potent and durable ZIKV-specific CD4^+^ and CD8^+^ T cells, Tfh cells, and neutralizing antibodies. This more balanced T and B cell activation represents a promising outcome of this combined vaccination strategy for clinical studies on ZIKV. Currently, the heterologous combination of SARS-CoV-2 vaccines is gaining support to further enhance the immunogenicity over the homologous vaccine administration that is being applied as a mass vaccination campaign to the human population. Understanding the B and T cell immune responses triggered by combined vaccines is needed for better implementation of worldwide vaccination programs.

## References

[1] Saiz JC, Vázquez-Calvo Á, Blázquez AB, et al. Zika virus: the latest newcomer. Front Microbiol. 2016;7:496. 27148186 10.3389/fmicb.2016.00496 PMC4835484

[2] Pielnaa P, Al-Saadawe M, Saro A, et al. Zika virus-spread, epidemiology, genome, transmission cycle, clinical manifestation, associated challenges, vaccine and antiviral drug development. Virology. 2020;543:34–42. 32056845 10.1016/j.virol.2020.01.015

[3] Agarwal A, Chaurasia D. The expanding arms of Zika virus: an updated review with recent Indian outbreaks. Rev Med Virol. 2021;31(1):1–9. 10.1002/rmv.2145 33216418

[4] Ryan SJ, Carlson CJ, Tesla B, et al. Warming temperatures could expose more than 1.3 billion new people to Zika virus risk by 2050. Glob Chang Biol. 2021;27(1):84–93. 33037740 10.1111/gcb.15384 PMC7756632

[5] Dowd KA, Ko S-Y, Morabito KM, et al. Rapid development of a DNA vaccine for Zika virus. Science. 2016;354(6309):237–240. 27708058 10.1126/science.aai9137 PMC5304212

[6] Pattnaik A, Sahoo BR, Pattnaik AK. Current status of Zika virus vaccines: successes and challenges. Vaccines. 2020;8(2):266. 10.3390/vaccines8020266 PMC7349928 32486368

[7] García-Arriaza J, Garaigorta U, Pérez P, et al. COVID-19 vaccine candidates based on modified vaccinia virus Ankara expressing the SARS-CoV-2 spike induce robust T- and B-cell immune responses and full efficacy in mice. J Virol. 2021;95(7):e02260–20. 10.1128/JVI.02260-20 PMC8092708 33414159

[8] Gómez CE, Nájera JL, Perdiguero B, et al. The HIV/AIDS vaccine candidate MVA-B administered as a single immunogen in humans triggers robust, polyfunctional, and selective effector memory T cell responses to HIV-1 antigens. J Virol. 2011;85(21):11468–11478. 21865377 10.1128/JVI.05165-11 PMC3194965

[9] Volz A, Sutter G. Modified vaccinia virus Ankara: history, value in basic research, and current perspectives for vaccine development. Adv Virus Res. 2017;97:187–243. 28057259 10.1016/bs.aivir.2016.07.001 PMC7112317

[10] Price PJR, Torres-Domínguez LE, Brandmüller C, et al. Modified vaccinia virus Ankara: innate immune activation and induction of cellular signalling. Vaccine. 2013;31(39):4231–4234. 23523404 10.1016/j.vaccine.2013.03.017

[11] Delaloye J, Roger T, Steiner-Tardivel Q-G, et al. Innate immune sensing of modified vaccinia virus Ankara (MVA) is mediated by TLR2-TLR6, MDA-5 and the NALP3 inflammasome. PLoS Pathog. 2009;5(6):e1000480. 19543380 10.1371/journal.ppat.1000480 PMC2691956

[12] Pérez P, Marín MQ, Lázaro-Frías A, et al. A vaccine based on a modified vaccinia virus Ankara vector expressing Zika virus structural proteins controls Zika virus replication in mice. Sci Rep. 2018;8(1):17385. 30478418 10.1038/s41598-018-35724-6 PMC6255889

[13] Yang Z-y, Wyatt LS, Kong W-p, et al. Overcoming immunity to a viral vaccine by DNA priming before vector boosting. J Virol. 2003;77(1):799–803. 12477888 10.1128/JVI.77.1.799-803.2003 PMC140625

[14] Li S, Rodrigues M, Rodriguez D, et al. Priming with recombinant influenza virus followed by administration of recombinant vaccinia virus induces CD8+ T-cell-mediated protective immunity against malaria. Proc Natl Acad Sci USA. 1993;90(11):5214–5218. 7685119 10.1073/pnas.90.11.5214 PMC46686

[15] Marín MQ, Pérez P, Ljungberg K, et al. Potent anti-hepatitis C virus (HCV) T cell immune responses induced in mice vaccinated with DNA-launched RNA replicons and modified vaccinia virus Ankara-HCV. J Virol. 2019;93(7):e00055–19. 30674625 10.1128/JVI.00055-19 PMC6430543

[16] Lorenzo G, López-Gil E, Ortego J, et al. Efficacy of different DNA and MVA prime-boost vaccination regimens against a rift valley fever virus (RVFV) challenge in sheep 12 weeks following vaccination. Vet Res. 2018;49(1):21. 29467018 10.1186/s13567-018-0516-z PMC5822472

[17] Gray GE, Mayer KH, Elizaga ML, et al. Subtype C gp140 vaccine boosts immune responses primed by the South African AIDS vaccine initiative DNA-C2 and MVA-C HIV vaccines after more than a 2-year Gap. Clin Vaccine Immunol. 2016;23(6):496–506. 27098021 10.1128/CVI.00717-15 PMC4895009

[18] Joachim A, Bauer A, Joseph S, et al. Boosting with subtype C CN54rgp140 protein adjuvanted with glucopyranosyl lipid adjuvant after priming with HIV-DNA and HIV-MVA is safe and enhances immune responses: A phase I trial. PLoS One. 2016;11(5):e0155702. 27192151 10.1371/journal.pone.0155702 PMC4871571

[19] Swadling L, Capone S, Antrobus RD, et al. A human vaccine strategy based on chimpanzee adenoviral and MVA vectors that primes, boosts, and sustains functional HCV-specific T cell memory. Sci Transl Med. 2014;6(261):261ra153. 10.1126/scitranslmed.3009185 PMC4669853 25378645

[20] Mensah VA, Gueye A, Ndiaye M, et al. Safety, immunogenicity and efficacy of prime-boost vaccination with ChAd63 and MVA encoding ME-TRAP against plasmodium falciparum infection in adults in Senegal. PLoS One. 2016;11(12):e0167951. 27978537 10.1371/journal.pone.0167951 PMC5158312

[21] Ewer K, Rampling T, Venkatraman N, et al. A monovalent Chimpanzee Adenovirus ebola vaccine boosted with MVA. N Engl J Med. 2016;374(17):1635–1646. 25629663 10.1056/NEJMoa1411627 PMC5798586

[22] Green CA, Scarselli E, Sande CJ, et al. Chimpanzee adenovirus- and MVA-vectored respiratory syncytial virus vaccine is safe and immunogenic in adults. Sci Transl Med. 2015;7(300):300ra126. 10.1126/scitranslmed.aac5745 PMC4669850 26268313

[23] Li Y, Bi Y, Xiao H, et al. A novel DNA and protein combination COVID-19 vaccine formulation provides full protection against SARS-CoV-2 in rhesus macaques. Emerg Microbes Infect. 2021;10(1):342–355. 33555988 10.1080/22221751.2021.1887767 PMC7928010

[24] He Q, Mao Q, An C, et al. Heterologous prime-boost: breaking the protective immune response bottleneck of COVID-19 vaccine candidates. Emerg Microbes Infect. 2021;10(1):629–637. 33691606 10.1080/22221751.2021.1902245 PMC8009122

[25] Vázquez-Calvo Á, Blázquez A-B, Escribano-Romero E, et al. Zika virus infection confers protection against West Nile virus challenge in mice. Emerg Microbes Infect. 2017;6:e81. 28928416 10.1038/emi.2017.68 PMC5625318

[26] Blázquez AB, Escribano-Romero E, Merino-Ramos T, et al. Infection with Usutu virus induces an autophagic response in mammalian cells. PLoS Negl Trop Dis. 2013;7:e2509. 24205422 10.1371/journal.pntd.0002509 PMC3812092

[27] Merino-Ramos T, Blázquez A-B, Escribano-Romero E, et al. Protection of a single dose West Nile virus recombinant subviral particle vaccine against lineage 1 or 2 strains and analysis of the cross-reactivity with Usutu virus. PLoS One. 2014;9:e108056. 25229345 10.1371/journal.pone.0108056 PMC4168257

[28] Martin-Acebes MA, Merino-Ramos T, Blazquez A-B, et al. The composition of West Nile virus lipid envelope unveils a role of sphingolipid metabolism in flavivirus biogenesis. J Virol. 2014;88(20):12041–12054. 25122799 10.1128/JVI.02061-14 PMC4178726

[29] García-Arriaza J, Nájera JL, Gómez CE, et al. A candidate HIV/AIDS vaccine (MVA-B) lacking vaccinia virus gene C6L enhances memory HIV-1-specific T-cell responses. PLoS One. 2011;6(8):e24244. 21909386 10.1371/journal.pone.0024244 PMC3164197

[30] García-Arriaza J, Arnáez P, Gómez CE, et al. Improving adaptive and memory immune responses of an HIV/AIDS vaccine candidate MVA-B by Deletion of vaccinia virus genes (C6L and K7R) blocking Interferon signaling pathways. PLoS One. 2013;8(6):e66894. 23826170 10.1371/journal.pone.0066894 PMC3694958

[31] Garcia-Arriaza J, Gomez CE, Sorzano COS, et al. Deletion of the vaccinia virus N2L gene encoding an inhibitor of IRF3 improves the immunogenicity of modified vaccinia virus Ankara expressing HIV-1 antigens. J Virol. 2014;88:3392–3410. 24390336 10.1128/JVI.02723-13 PMC3957918

[32] Pérez P, Marín MQ, Lázaro-Frías A, et al. An MVA vector expressing HIV-1 envelope under the control of a potent vaccinia virus promoter as a promising strategy in HIV/AIDS vaccine design. Vaccines. 2019;7:208. 10.3390/vaccines7040208 PMC6963416 31817622

[33] Perdiguero B, Gómez CE, García-Arriaza J, et al. Heterologous combination of VSV-GP and NYVAC vectors expressing HIV-1 trimeric gp145 Env as vaccination strategy to induce balanced B and T cell immune responses. Front Immunol. 2019;10:2941. 31921191 10.3389/fimmu.2019.02941 PMC6930178

[34] Perdiguero B, Raman SC, Sánchez-Corzo C, et al. Potent HIV-1-specific CD8 T cell responses induced in mice after priming with a multiepitopic DNA-TMEP and boosting with the HIV vaccine MVA-B. Viruses. 2018;10(8):424. 10.3390/v10080424 PMC6116222 30104537

[35] García-Arriaza J, Nájera JL, Gómez CE, et al. Immunogenic profiling in mice of a HIV/AIDS vaccine candidate (MVA-B) expressing four HIV-1 antigens and potentiation by specific gene deletions. PLoS One. 2010;5(8):e12395. 20811493 10.1371/journal.pone.0012395 PMC2927552

[36] Lorenz IC, Kartenbeck J, Mezzacasa A, et al. Intracellular assembly and secretion of recombinant subviral particles from tick-borne encephalitis virus. J Virol. 2003;77(7):4370–4382. 12634393 10.1128/JVI.77.7.4370-4382.2003 PMC150630

[37] Wen J, Elong Ngono A, Angel Regla-Nava J, et al. Dengue virus-reactive CD8+ T cells mediate cross-protection against subsequent Zika virus challenge. Nat Commun. 2017;8(1):1459. 29129917 10.1038/s41467-017-01669-z PMC5682281

[38] Nazerai L, Schøller AS, Bassi MR, et al. Effector CD8 T cell-dependent Zika virus control in the CNS: a matter of time and numbers. Front Immunol. 2020;11:1977. 32973802 10.3389/fimmu.2020.01977 PMC7461798

[39] Crotty S. T follicular helper cell differentiation, function, and roles in disease. Immunity. 2014;41(4):529–542. 25367570 10.1016/j.immuni.2014.10.004 PMC4223692

[40] Reinhardt RL, Liang HE, Locksley RM. Cytokine-secreting follicular T cells shape the antibody repertoire. Nat Immunol. 2009;10(4):385–393. 19252490 10.1038/ni.1715 PMC2714053

[41] Larocca RA, Abbink P, Peron JPS, et al. Vaccine protection against Zika virus from Brazil. Nature. 2016;536(7617):474–478. 27355570 10.1038/nature18952 PMC5003703

[42] Abbink P, Larocca RA, De La Barrera RA, et al. Protective efficacy of multiple vaccine platforms against Zika virus challenge in rhesus monkeys. Science. 2016;353(6304):1129–1132. 27492477 10.1126/science.aah6157 PMC5237380

[43] Betancourt D, de Queiroz NMGP, Xia T, et al. Cutting edge: Innate immune augmenting vesicular stomatitis virus expressing Zika virus proteins confers protective immunity. J Immunol. 2017;198(8):3023–3028. 28289159 10.4049/jimmunol.1602180

[44] Nürnberger C, Bodmer BS, Fiedler AH, et al. A measles virus-based vaccine candidate mediates protection against Zika virus in an allogeneic mouse pregnancy model. J Virol. 2019;93(3):e01485–18. 30429338 10.1128/JVI.01485-18 PMC6340036

[45] Zhan Y, Deng Y, Huang B, et al. Humoral and cellular immunity against both ZIKV and poxvirus is elicited by a two-dose regimen using DNA and non-replicating vaccinia virus-based vaccine candidates. Vaccine. 2019;37(15):2122–2130. 30851967 10.1016/j.vaccine.2019.02.063

[46] López-Camacho C, Kim YC, Abbink P, et al. Assessment of immunogenicity and efficacy of a zika vaccine using modified vaccinia Ankara virus as carriers. Pathogens. 2019;8(4):216. 10.3390/pathogens8040216 PMC6963679 31684117

[47] Prow NA, Liu L, Nakayama E, et al. A vaccinia-based single vector construct multi-pathogen vaccine protects against both Zika and chikungunya viruses. Nat Commun. 2018;9(1):1230. 29581442 10.1038/s41467-018-03662-6 PMC5964325

[48] Brault AC, Domi A, McDonald EM, et al. A Zika vaccine targeting NS1 protein protects immunocompetent adult mice in a lethal challenge model. Sci Rep. 2017;7(1):14769. 29116169 10.1038/s41598-017-15039-8 PMC5677088

[49] Ngono AE, Shresta S. Immune response to Dengue and Zika. Annu Rev Immunol. 2018;36:279–308. 29345964 10.1146/annurev-immunol-042617-053142 PMC5910217

[50] Yau C, Gan ES, Kwek SS, et al. Live vaccine infection burden elicits adaptive humoral and cellular immunity required to prevent Zika virus infection. EBioMedicine. 2020;61:103028. 33045466 10.1016/j.ebiom.2020.103028 PMC7553235

[51] Pardy RD, Richer MJ. Protective to a T: the role of T cells during Zika virus infection. Cells. 2019;8(8):820. 10.3390/cells8080820 PMC6721718 31382545

[52] Gaudinski MR, Houser KV, Morabito KM, et al. Safety, tolerability, and immunogenicity of two Zika virus DNA vaccine candidates in healthy adults: randomised, open-label, phase 1 clinical trials. Lancet. 2018;391(10120):552–562. 29217376 10.1016/S0140-6736(17)33105-7 PMC6379903

[53] Steffen T, Hassert M, Hoft SG, et al. Immunogenicity and efficacy of a recombinant human adenovirus type 5 vaccine against Zika virus. Vaccines. 2020;8(2):170. 10.3390/vaccines8020170 PMC7349816 32272595

[54] Hassert M, Harris MG, Brien JD, et al. Identification of protective CD8 T cell responses in a mouse model of Zika virus infection. Front Immunol. 2019;10:1678. 31379867 10.3389/fimmu.2019.01678 PMC6652237

[55] Guo Q, Chan JF-W, Poon VK-M, et al. Immunization with a novel human type 5 adenovirus-vectored vaccine expressing the premembrane and envelope proteins of Zika virus provides consistent and sterilizing protection in multiple immunocompetent and immunocompromised animal models. J Infect Dis. 2018;218(3):365–377. 29617816 10.1093/infdis/jiy187

[56] Bullard BL, Corder BN, Gorman MJ, et al. Efficacy of a T cell-biased Adenovirus vector as a Zika virus vaccine. Sci Rep. 2018;8(1):18017. 30573745 10.1038/s41598-018-35755-z PMC6301965

[57] Seder RA, Darrah PA, Roederer M. T-cell quality in memory and protection: implications for vaccine design. Nat Rev Immunol. 2008;8(4):247–258. 18323851 10.1038/nri2274

[58] Samy N, Reichhardt D, Schmidt D, et al. Safety and immunogenicity of novel modified vaccinia Ankara-vectored RSV vaccine: a randomized phase I clinical trial. Vaccine. 2020;38(11):2608–2619. 32057576 10.1016/j.vaccine.2020.01.055

[59] García F, Bernaldo de Quirós JCL, Gómez CE, et al. Safety and immunogenicity of a modified pox vector-based HIV/AIDS vaccine candidate expressing Env, Gag, Pol and Nef proteins of HIV-1 subtype B (MVA-B) in healthy HIV-1-uninfected volunteers: A phase I clinical trial (RISVAC02). Vaccine. 2011;29(46):8309–8316. 21907749 10.1016/j.vaccine.2011.08.098

[60] Tebas P, Roberts CC, Muthumani K, et al. Safety and immunogenicity of an anti–Zika virus DNA vaccine – preliminary report. N Engl J Med. 2017. doi:10.1056/NEJMoa1708120. PMC6824915 34525286

